# Identification of a Hypoxia-Angiogenesis lncRNA Signature Participating in Immunosuppression in Gastric Cancer

**DOI:** 10.1155/2022/5209607

**Published:** 2022-08-23

**Authors:** Zicheng Wang, Xisong Liang, Hao Zhang, Zeyu Wang, Xun Zhang, Ziyu Dai, Zaoqu Liu, Jian Zhang, Peng Luo, Jiarong Li, Quan Cheng

**Affiliations:** ^1^Hepatobiliary Surgery, Hunan Provincial People's Hospital (The First Affiliate Hospital of Hunan Normal University), Changsha, China; ^2^Department of Neurosurgery, Xiangya Hospital, Central South University, Changsha 410008, China; ^3^National Clinical Research Center for Geriatric Disorders, Xiangya Hospital, Central South University, Changsha 410008, China; ^4^Department of Interventional Radiology, The First Affiliated Hospital of Zhengzhou University, Zhengzhou, Henan, China; ^5^Department of Oncology, Zhujiang Hospital, Southern Medical University, Guangzhou 510000, China; ^6^Department of General Surgery, Xiangya Hospital, Central South University, Changsha 410008, China

## Abstract

Hypoxia and angiogenesis are the leading causes of tumor progression, and their strong correlation has been discovered in many cancers. However, their collective function's prognostic and biological roles were not reported in gastric cancer. Hence, we aimed to investigate the effects of hypoxia and angiogenesis on gastric cancer via sequencing data. This study used weighted gene coexpression network analysis and random forest regression to build a hypoxia-angiogenesis-related model (HARM) via the TCGA-STAD lncRNA data. It estimated the HARM's correlation with clinical features and its accuracy for survival prediction. Sequential functional analyses were conducted to investigate its biological role, and we next sought the immune landscape status and immunological function variation by ESTIMATE score calculation and GSVA, respectively. Seven different algorithms were conducted to assess the immunocyte infiltration, and TIDE score and immune checkpoint levels were compared between the high- and low-HARM groups. As a result, we found that HARM predicted patient survival with high accuracy and was correlated with higher stages of gastric cancer. Various cancer-associated pathways and macrophage-related regulations were upregulated in the high-HRAM group. The high-HARM group harbored higher immune levels, and M2 macrophages and cancer-associated fibroblasts were particularly highly unfiltered. Furthermore, globally upregulated immune checkpoints and higher TIDE scores were observed in the high-HARM group. Finally, we filtered eight drugs with lower IC50 in the high-HARM group as potential drugs for the HARM-targeted therapy. We believe this study opens up novel perspectives into the interaction between hypoxia-angiogenesis and immunosuppression and will provide novel insights for gastric cancer immunotherapy.

## 1. Introduction

Gastric carcinoma (GC) is one of the most common malignant tumors of the digestive tract with high incidence and fatality rates. More than 783,000 people die of gastric cancer every year [1, 2]. Most GC patients were first diagnosed with advanced stages, suffering from the limited therapeutic effects of routine treatment, including surgery, radiotherapy, and chemotherapy [3]. Hence, novel therapeutic strategies are urgently required.

Molecular therapy is an effective approach [4] and has recently been proved promising for treating GC patients. The emerging gene targets, including HER2, EGFR, VEGF, MET, and mTOR, were tested in clinical trials. Still, only HER2 and microsatellite instability (MSI) were successfully applied to clinical practice, and not all patients benefited from them [5]. Numerous efforts are still made to seek novel and effective oncogene targets; these oncogenes participate in various biological activities of gastric cancer, such as drug resistance and ubiquitination [6, 7]. Among the various biological activities, hypoxia and angiogenesis were the leading risky causes to support GC progress [8, 9, 10, 11, 12]. Angiogenesis can be activated by cytokines and promote GC cell metastasis [12]; hypoxia induces the long noncoding RNA (lncRNA) expression and support GC cell proliferation and metastasis [11]. Importantly, hypoxia showed a strong mutual correlation with GC angiogenesis. For instance, VEGF has been found to be highly expressed in GC cancer cells under hypoxia [9], and activation of HIF-1*α* promoted angiogenesis [8]. Similarly, HIF-1*α* downregulation inhibited angiogenesis [10]. Given the high interaction between angiogenesis and hypoxia, strategies targeting their cooperation should be developed, but no such work has been reported.

lncRNAs with a length of more than 200 nucleotides have been proved to affect cancer fate [13, 14] and have been noticed as risky factors contributing to GC progress [15, 16, 17], as well as other cancers [14]. They enhanced tumorigenesis via HIF-1*α* modulation [17], promoted angiogenesis via VEGFR2 signaling pathway activation [18], and influenced GC cell's proliferation, migration, and invasion [15]. To date, lncRNA-signature prediction models were found to predict GC patient survival, including an angiogenesis-related signature [19], while no model concerning hypoxia was reported.

Environment immunity plays a critical but dual role in GC cell progression [20]. Immunocytes attack cancer cells once they are identified, while they can escape from immune surveillance by destroying normal immunocytes or promoting their protumor polarization [21]. During cancer development, immune checkpoints were the predominant targets of the immunosuppressive environment formation [22], and immune checkpoint therapy has been validated as an effective strategy, but how many immune checkpoints function in GC remained unclear [23]. For the causes of immunosuppression, hypoxia decreased macrophage M1 percentage and HIF-1*α* promoted their M2 polarization [24, 25]; integrative therapy targeting both VEGF and immune checkpoints has presented promising effects [26]. Therefore, hypoxia and angiogenesis showed a high correlation with cancer immunity. In contrast, comprehensive analyses of hypoxia, angiogenesis, and immune in GC are still lacking.

**Figure 1:**
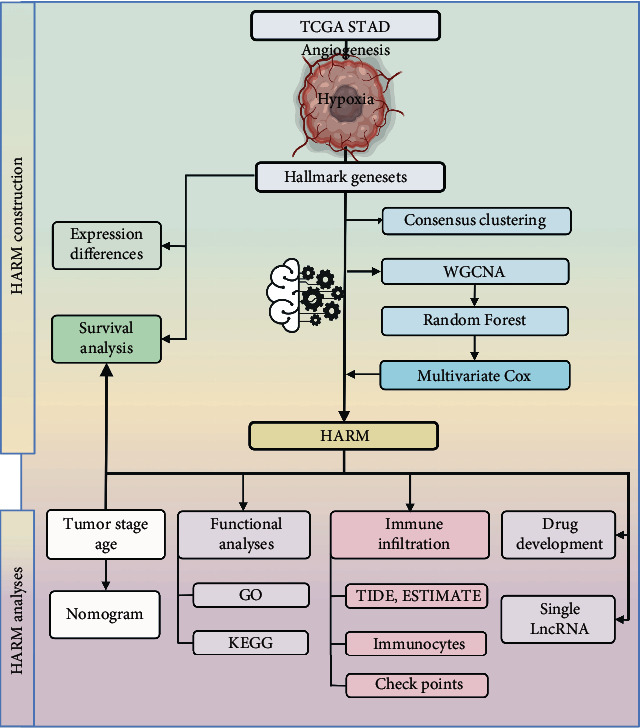
The workflow of this study. The hypoxia-angiogenesis-related lncRNA was obtained to construct a HARM model by WGCNA, Random Forest regression, and multivariate Cox analysis. The clinical significance of the HARM was analyzed, and the functional analyses were conducted to detect its biological engagement in GC. A series of immune analyzing approaches were applied to investigate the immunological diversity of the HARM groups. Finally, the HARM-sensitive drugs were screened out, and the single lncRNA's clinical significance in the HARM was investigated.

In this study, we constructed a robust hypoxia-angiogenesis-related lncRNA model (HARM) by weighted gene coexpression network analysis (WGCNA), random forest, and multivariate Cox regression. We validated its predictive ability for patient survival. The association between the HARM and the immune was comprehensively explored by immune scores, immunocyte infiltration, immunological function, and immune checkpoint analyses. Finally, the sensitivities of potential HALM-targeted drugs were calculated (Figure 1). We believe this study will shed light on the immunological roles of hypoxia and angiogenesis in GC and benefit patients by providing novel therapeutic strategies.

## 2. Materials and Methods

### 2.1. Gene sets and Dataset Collection

The gene sets for prognostic parameter selection were collected from the “Hallmark gene sets” from the Molecular Signatures Database (MSigDB) of Gene Set Enrichment Analysis (GSEA). The samples with sequence data and clinical information used in this study were obtained from TCGA-STAD dataset, and their clinical data is exhibited in Table 1.

### 2.2. Selection of Survival-Related Gene Sets and Assessment of Their Prognostic Significance

We first collected the “hallmark gene sets” from the MSigDB of the GSEA, and we conducted a univariate cox analysis to calculate their hazard ratio to select the significantly risky gene sets for GC patients. The levels of the selected risky gene sets were then compared between alive and dead patients. Subsequently, the patients were divided into the high-level and low-level groups, and survival analyses were conducted between the high- and low-level groups to estimate their prognostic significance.

### 2.3. Consensus Clustering and Its Association with Survival and Risky Gene Sets

To obtain the samples' unsupervised clustering results, we ran the Consensus Clustering, an unsupervised clustering method to cluster samples according to their intrinsic transcriptional features, using the R package “ConsensusClusterPlus” [27]. The cumulative distribution function (CDF) and the area under the CDF change plot were drawn to determine the k value of the clustering. The survival analysis was conducted to determine whether the clusters can separate patient survival probabilities, and the levels of risky gene sets were compared.

### 2.4. Weighted Gene Coexpression Network Analysis and Random Forest Model Construction

WGCNA of the risky gene sets was applied to filter the lncRNA firmly relevant to hypoxia and angiogenesis using the r package “WGCNA” [28], a package tool to select parameters firmly associated with the interested phenotypes of cancers based on parameters modules, and this was followed by a univariate cox analysis to further screen out the prognostic lncRNA with statistical significance.

Subsequently, we performed random forest, a machine learning regression approach to decrease the model's parameters with a better generalization than a single tree, by employing the randomForest function of the r package “randomforest,” and applied the multivariate Cox regression to construct the prediction model. The variable importance of each lncRNA was listed, and the candidate models were also presented in a bar chart. The top model with the lowest *P* value was selected as the final HARM model.

### 2.5. The Prognostic Significance Estimation of the HARM

According to the HARM score we constructed above, the patients were divided into the the low- and high-HARM groups. After grouping, we performed the survival analysis to clarify whether the HARM can predict the survival of the GC patients. We then present the HARM predictors' expression of the samples in a heatmap to observe the consistency between the lncRNA and the HARM. Moreover, we ran a 3D principal clustering analysis (PCA) visualization of the HARM predictor and compared the spatial distribution of the low and high HARM scores in the clustered samples. The receiver operating characteristic (ROC) curve and *c*-index were applied to assess the discrimination of the HARM for predicting 1-, 3-, and 5-year survival and compare the predicting ability between HARM and age, gender, grade, and stage, respectively. The age, gender, grade, stage, and HARM hazard ratio were also exhibited in the forest plot. Moreover, we compared the mutual association between risks and clusters in GC samples according to the levels of one classifier within the groups of the other.

### 2.6. Clinical Relevance of the HARM

We calculated the HARM score of samples divided by stage, TNM, gender, and age groups to explore whether the HARM was correlated with GC clinical features. The age higher than 60 was defined as the “>60” group, and the remaining was divided into “≤60” group. Additionally, we compared the prognostic ability of HARM for patients stratified by stage, gender, and age. Finally, a clinical nomogram integrating age, stage, and the HARM was established, and its predictive capabilities for GC patients 1-, 3-, and 5-year overall survival (OS) were evaluated by ROC and calibration curves.

### 2.7. Functional Enrichment Analyses of Differentially Expressed Genes between HARM Groups

We conducted a series of functional enrichment analyses to investigate the biological roles of HARM in GC patients. The GSEA analyses [29] of gene sets from biological processes of Gene Ontology (GO) and Kyoto Encyclopedia of Genes and Genomes (KEGG) for differentially enriched gene sets in the high-HARM group were, respectively, conducted. Gene set variation analysis (GSVA) of the GO and KEGG genes was also performed to show the differentially variated gene sets [30].

### 2.8. Immunological Role of HARM in GC Patients

To seek whether the HARM interplayed with immunity in the GC microenvironment, we firstly compared the global immune landscape between the low- and high-HARM groups by analyzing ESTIMATEScore, ImmuneScore, and StromalScore [31]. To go further, we calculated the immunocyte infiltration by seven different algorithms (TIMER, CIBERSORT, CIBERSORT-ABS, QIAMTISEQ, MCPCOUNTER, XCELL, and EPIC) [32] and exhibited the infiltrating diversity of each immunocyte between the low- and high-HARM groups. The immune-related pathways were also enriched for each sample and presented by a heatmap [30]. Finally, the TIDE scores [33] and immune checkpoint gene expressions were compared between the HARM groups [34].

### 2.9. Investigation of Drug Sensitivity Differences between Risk Groups

For developing potential drugs targeting the HARM signature, we calculated the 50% inhibitory concentration (IC50) of the GDSC anticancer drugs against various cell lines using the r package “pRRophetic” [35]. The IC50 was compared between the low- and high-HARM groups for each drug.

### 2.10. Clinical Significance of the Single lncRNA in the HARM

We adopted GEPIA2.0, (http://gepia2.cancer-pku.cn/), a web tool for investigating the comprehensive information of human genes, to compare the expression differences of the single lncRNA in the HARM between cancer and noncancer tissues. Besides, we analyzed single lncRNA's prognostic significance and its association between expression and stages.

### 2.11. Statistical Analyses

For differences in comparison between the two groups, Student's *t*-test was used for normally distributed parameters, and Wilcoxon test was used for nonnormally distributed parameters. Univariate cox analysis was conducted to select survival-associated parameters. Correlations were calculated using Pearson's correlation coefficients. The discrimination of the model predictive ability was estimated using ROC. *χ*^2^ test was applied to test the percentage differences. A one-way ANOVA tested grouped analyses for normally distributed values, and a nonparametric Kruskal-Wallis test was applied for nonnormally distributed values of the grouped test. *P* values less or equal to 0.05 were considered statistically significant.

## 3. Results

### 3.1. Hypoxia and Angiogenesis in GC Were Associated with Poor Prognosis

**Figure 2:**
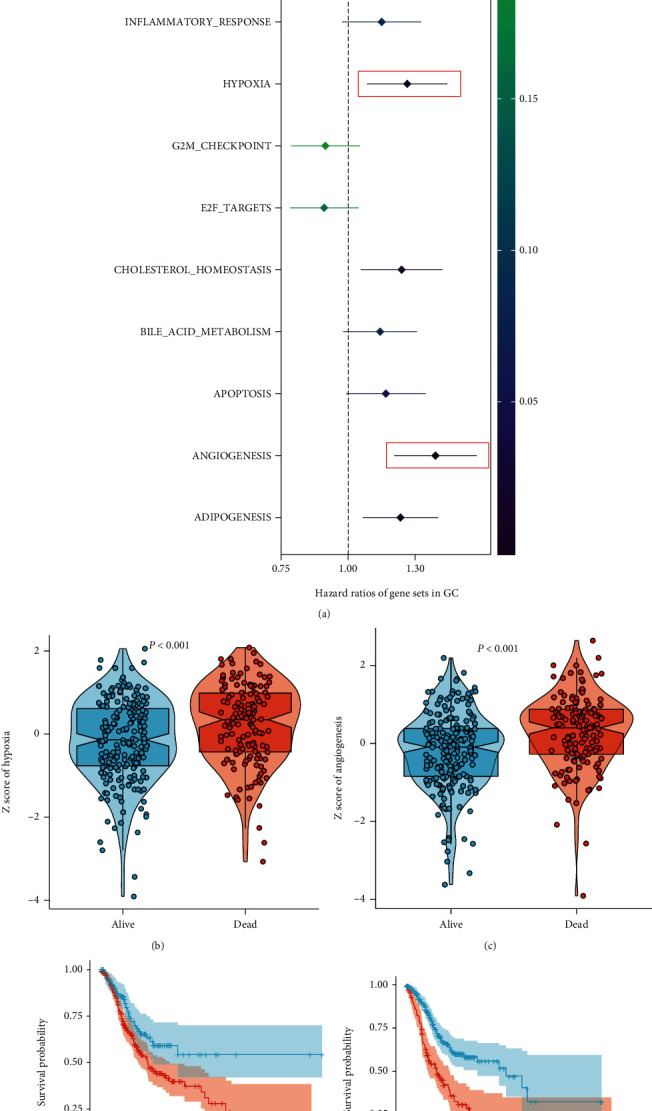
The selection of survival-related gene sets. (a) The forest plot shows the hazard ratios of the “Hallmark gene sets”; the red box represents the two gene sets with the highest hazard ratios. The box plot exhibits the expression differences of (b) hypoxia and (c) angiogenesis between alive and dead patients. The ability of (d) hypoxia and (e) angiogenesis signatures to separate the survival risks of patients presented by Kaplan-Meier plots.

The “hallmark gene set” collected from MSigDB of GSEA was filtered by univariate cox analysis. We found that hypoxia and angiogenesis were the most significant risk factors for predicting overall survival (Figure 2(a)). The hypoxia and angiogenesis levels were higher in the dead group than in the alive group (Figures 2(b) and 2(c)). Moreover, patients with elevated hypoxia and angiogenesis levels exhibited worse survival probability as the Kaplan-Meier curves presented (Figures 2(d) and 2(e)). These suggested that hypoxia and angiogenesis in GC were closely related to patient survival.

### 3.2. Consensus Clustering, WGCNA, and Random Forest Model Construction

**Figure 3:**
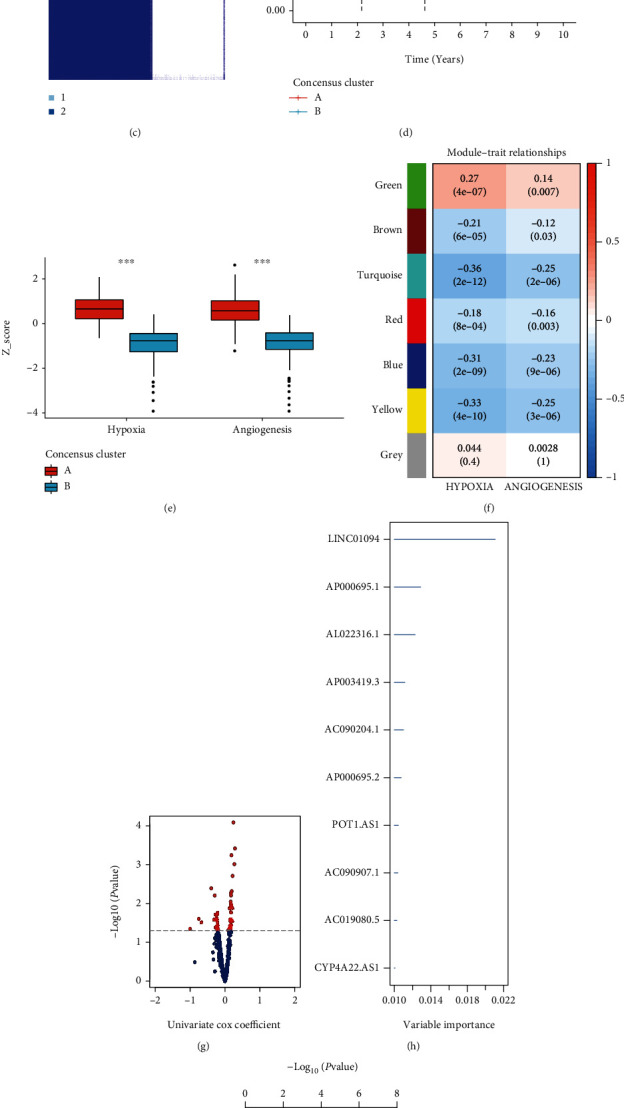
Consensus clustering, WGCNA, and random forest regression for model construction. The processes of consensus clustering show (a) CDF and (b) relative change in area under CDF curve for *k* = 2-9. (c) Heatmap of the patients' genetic expression pattern clustered by *k* = 2. (d) The ability to predict survival rates of consensus clusters is presented by Kaplan-Meier curves. (e) The box plot exhibits the expression differences of hypoxia and angiogenesis between the consensus clusters A and B. The WGCNA clustering modules of (f) gastric cancer and univariate cox analysis of the lncRNA selected from the (g) WGCNA module. (h) The bar chart listed the top 10 lncRNAs ranked by variable importance calculated by the random forest regression. (i) The best combination of the ten lncRNA was selected from multivariate Cox regression to build a prognostic model. ∗∗∗ represents the *P* value less than 0.001.

Unsupervised consensus clustering was applied to divide the GC samples into different clusters, and the samples presented apparent diversity when *k* = 2 (Figures 3(a)–3(c)). Survival analyses tested the prognostic value of the clusters, and the results showed that patients in cluster A exhibited worse overall survival than the patients in cluster B (Figure 3(d)). Besides, the boxplots showing the levels of hypoxia and angiogenesis were significantly elevated in cluster A (Figure 3(e)).

We then performed WGCNA analysis, divided the hypoxia and angiogenesis-related lncRNA into seven modules, and applied the critical module lncRNA to univariate cox analysis (Figures 3(f) and 3(g)). The survival-related lncRNAs were filtered under the statistically significant threshold (Figure 3(g)). Subsequently, the top 10 important lncRNA were selected by the random forest algorithm and applied to the multivariate Cox regression to build the HARM with the best combination. Finally, nine lncRNAs were determined for HARM construction (Figures 3(h) and 3(i)).

### 3.3. The HARM Predicted Survival and Hypoxia Angiogenesis Levels with High Accuracy

**Figure 4:**
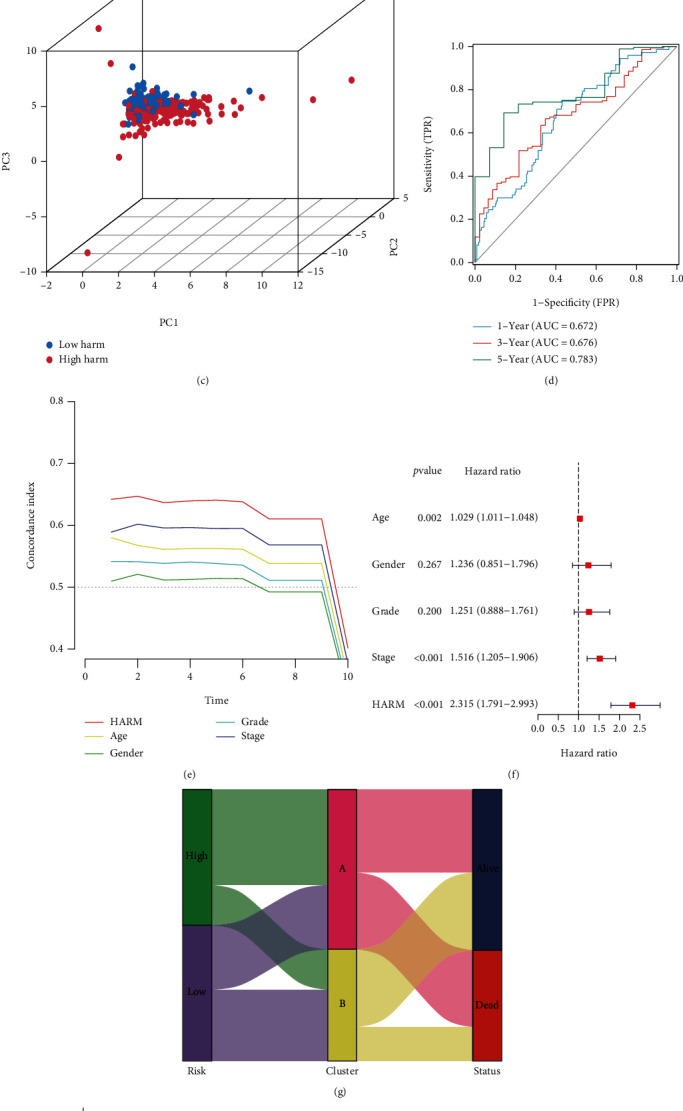
Estimation of the HARM prognostic values. (a) The Kaplan-Meier curves present the ability of HARM to separate patient survival. (b) The HARM levels of all gastric cancer samples and the expression of HARM lncRNAs in each patient were ranked by the HARM scores. (c) HARM level distribution in the samples visualized by 3D PCA. (d) ROC test exhibited the AUC value of the prediction ability for the HARM in predicting patients' 1-, 3-, and 5-year OS. (e) The comparison of the accuracy in predicting patient OS among HARM, age, gender, grade, and stage by *c*-index. (f) The forest plot exhibited the hazard ratios of age, gender, grade, stage, and HARM. (g) The Sankey diagram displayed the data flow from the HARM to clusters and survival status. (h) The violin plot compared the HARM sores between clusters A and B. (i) The bar chart compared the percentage of clusters A and B in the low- and high-HARM groups, respectively.

To validate the prognostic value of the HARM, we performed a survival analysis, and the HARM separated the survival of the GC patients remarkably as high HARM patients suffered lower survival rates (Figure 4(a)). The heatmap showed the expression of the nine lncRNA in the HARM score-ranked patients. CYP4A22-AS1 was highly expressed in the low-HARM group, while POT1-AS1 and LNC01094 presented the opposite trend (Figure 4(b)).  Figure 4(c) exhibits the clustering of the patients, and the high-HARM samples were apart from those with low-HARM. ROC and *c*-index results showed that the HARM performed well in predicting 1-, 3-, and 5-year OS and outperformed age, gender, grade, and stage (Figures 4(d)–4(f)). Additionally, we analyzed the HARM's correlation with the clusters. Most of the high-HARM data flowed to cluster A, as samples in cluster A harbored higher HARM scores. Most of the cluster A samples were included in the high-HARM group (Figures 4(g)–4(i)), demonstrating that HARM was a robust model with a higher prognostic value.

### 3.4. HARM Correlated with Higher Clinical Stages and Predicted Patient Survival Independently

**Figure 5:**
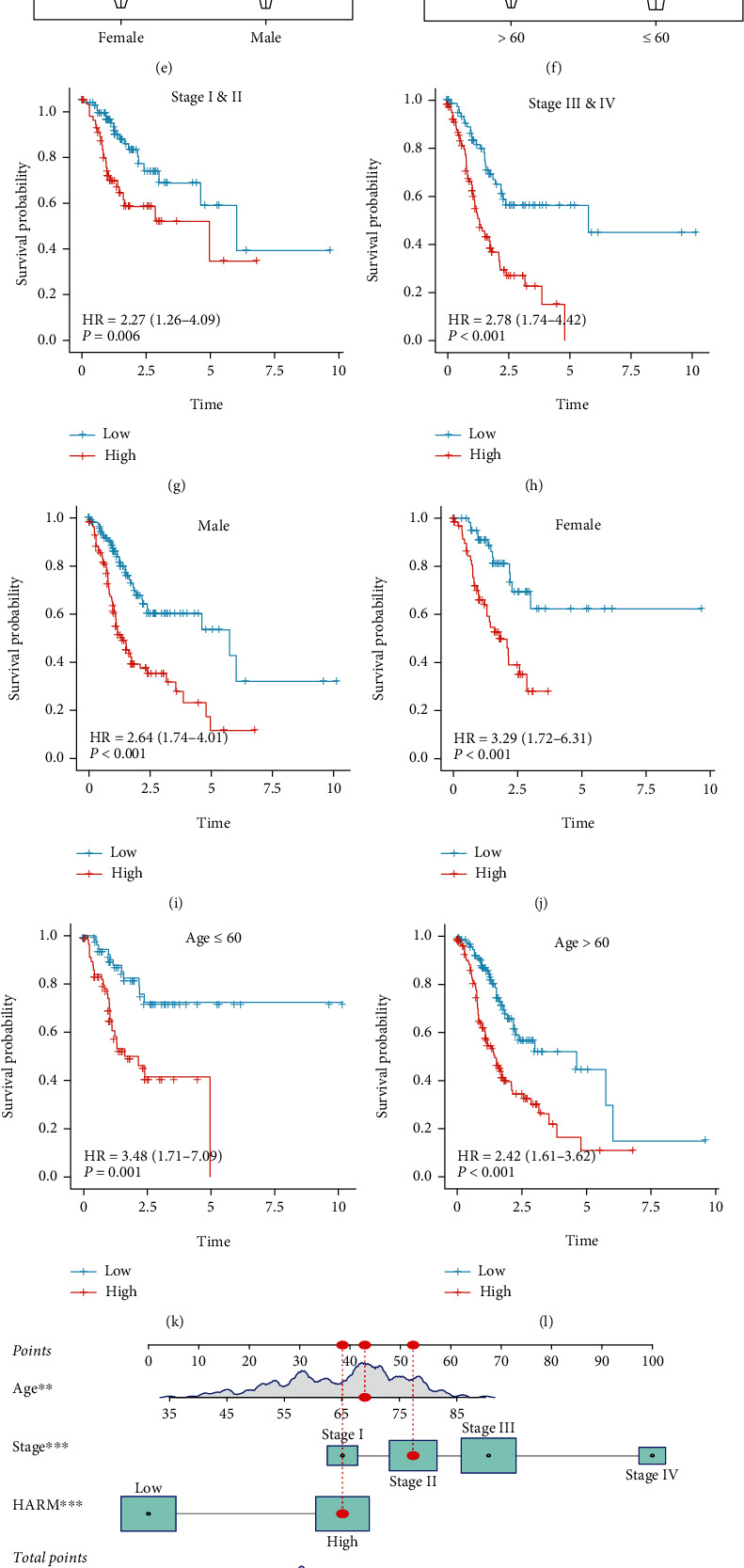
Clinical relevance of the HARM and its independent prognostic value. The violin plots displayed the HARM levels of patients in different stages (a), T stage (b), N stage (c), M stage (d), genders (e), and age groups (f). The Kaplan-Meier plots exhibited the ability of HARM to predict survival rates of patients in stages I and II (g), stages III and IV (h), male (i), female (j), age ≤ 60 (k), and age > 60 group (l). The nomogram was built by integrating age, stage, and the HARM (m), and its performance was estimated by ROC (n) and calibration curves (o). Ns means not statistically significant; ∗∗and∗∗∗ represent the *P* value less than 0.01 and 0.001, respectively.

To investigate HARM's correlation with clinical features, we compared the HARM scores among various clinical features, and the results showed that the HARM score increased with the TNM stage (Figures 5(a)–5(d)). However, no correlations were found between HARM and gender and age (Figures 5(e) and 5(f)). To seek whether the HARM can independently separate the prognosis of patients under different stages, gender, and age groups, we conducted survival analyses on them, and high HARM predicted poor prognosis in all these groups significantly (Figures 5(g)–5(l)). Finally, HARM, stage, and age, which were significantly associated with survival, were integrated to construct a clinical nomogram for predicting gastric cancer patients' survival (Figure 5(m)). The ROC and calibration curve demonstrated the high discrimination and calibration of the nomogram, respectively, when predicting 1-, 3-, and 5-year OS (Figures 5(n) and 5(o)). Shortly, these results indicated that the HARM can also predict the GC stage and survival independently and can present higher accuracy when integrated with common clinical features.

### 3.5. High HARM Was Associated with Immune, Cancer-Related Activities, and Pathways

**Figure 6:**
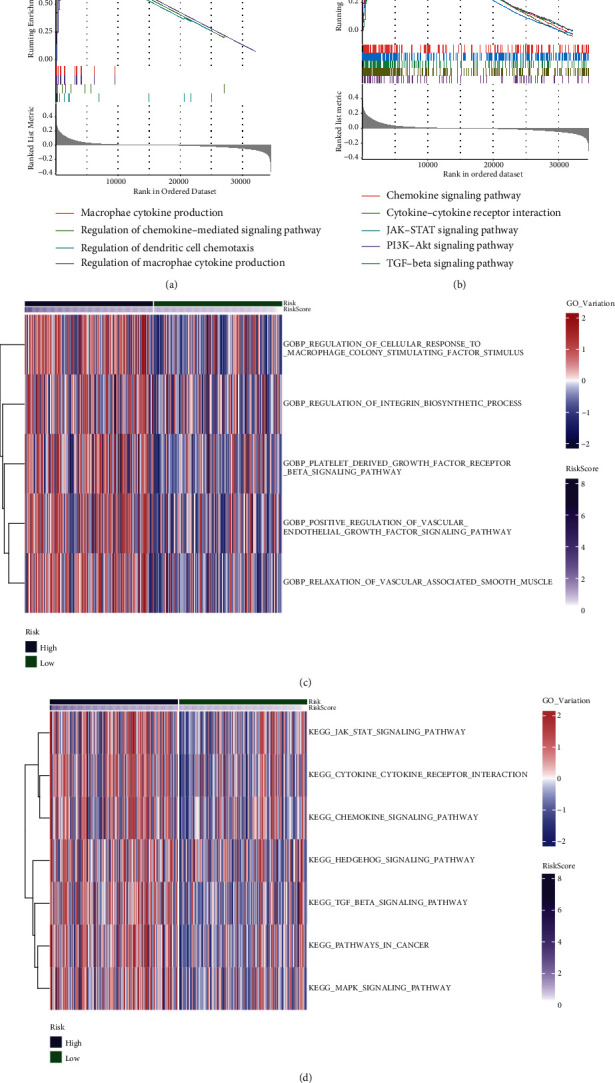
Functional enrichment of GO and KEGG datasets. The differentially expressed genes in the high-HARM group were analyzed by GO (a), KEGG (b) functional enrichment. Gene set variation analyses of the upregulated processes in GO (c) and KEGG (d) datasets.

To explore the role of HARM in the biological processes of gastric cancer, we performed functional enrichment analyses for differentially expressed genes (DEGs) between the high-and low-HARM groups. The enrichment results of GO database exhibited that immune-related biological processes and pathways were highly enriched in the high-HARM group, including chemokine-mediated signaling pathway, dendritic cell chemotaxis, and macrophage-associated activities (Figure 6(a)). For KEGG datasets, many cancer-related pathways are shown, such as JAK-STAT, PI3K-Akt, and TGF-*β* signaling pathways (Figure 6(b)). Besides, we performed GSVA in discovering highly variated pathways in GO and KEGG gene sets, and we noticed that GO biological processes of cellular response to macrophage-colony stimulating factor stimulus, integrin biosynthetic process, and vascular-related pathways were highly expressed in the high-HARM group (Figure 6(c)). In the KEGG dataset, JAK-STAT, TGF-*β*, MAPK, and other cancer-related pathways were upregulated in the high-HARM group (Figure 6(d)), similar to the GSEA results. The functional results suggested that hypoxia and angiogenesis might activate many cancer-associated pathways and affect the cancer immunity.

### 3.6. High-HARS GC Presented Higher Immunosuppressive Checkpoint Levels and Immunocyte Infiltration

**Figure 7:**
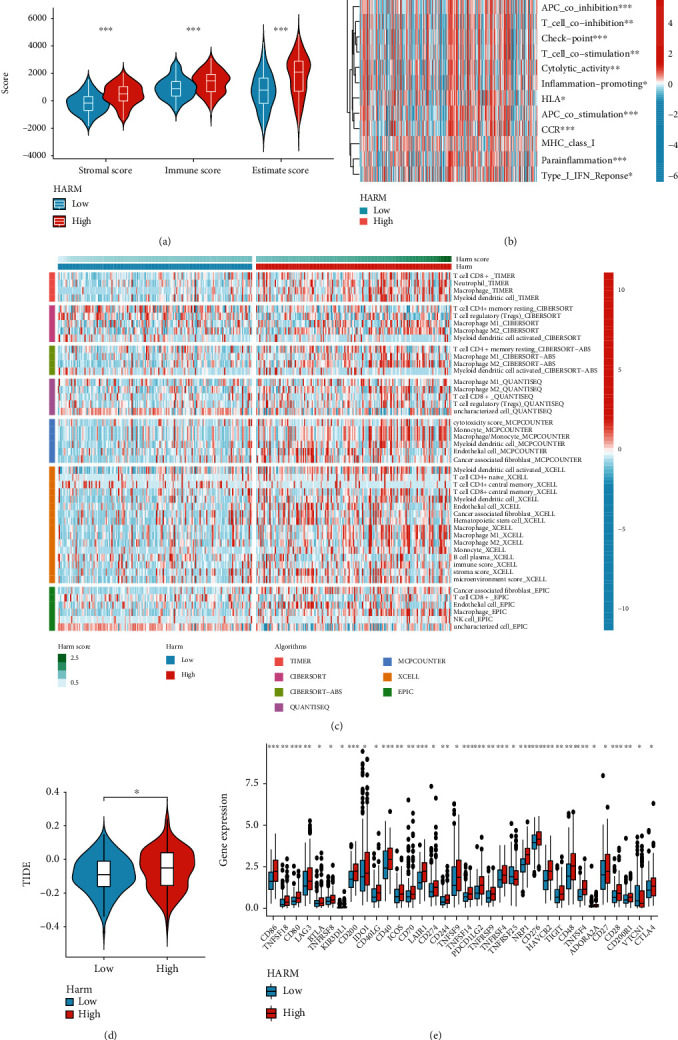
Investigation of immune diversity between the high-and low-HARM groups. (a) The violin plots exhibited the StromalScore, ImmuneScore, and ESTIMATEScore differences between the high- and low-HARM groups. (b) The heatmap shows the immune pathway variation between HARM groups by GSVA. (c) The immunocyte infiltration diversity between the two HARM groups was calculated using seven different algorithms and visualized in a heatmap. (d) The TIDE score levels were compared between the high-HARM and the low-HARME groups in a violin plot. (e) The box plots compare immune checkpoint expression levels between the high-and the low-HARM groups. Ns means not statistically significant; ∗, ∗∗, and∗∗∗ represent the *P* value less than 0.05, 0.01, and 0.001, respectively.

Since the HARS was suggested to affect immunity as the functional analyses presented above, we first compared the ESTIMATEScore, ImmuneScore, and StromalScore between the low-and high-HARM groups, and the results showed that the three scores were higher in the high-HARM group (Figures 7(a)). Further, the GSVA [30] analysis of the immune-related pathways was conducted, and the top 13 pathways were presented in a heatmap; the majority of them were highly enriched in the high-HARM group (Figure 7(b)).

Subsequently, we applied 7 different algorithms to compare the immunocyte infiltration levels between high-and low-HARM groups, and we noticed that high-HARM was associated with high immunocyte infiltration. Notably, M2 macrophages and cancer-associated fibroblasts (CAFs) infiltration were remarkably elevated in the high-HARM group (Figure 7(c)), which were immunosuppressive cells in the tumor microenvironment. The high levels of immunosuppressive cell infiltration arose the question whether the HARM can affect immunotherapy and whether immune checkpoints were highly expressed, to investigate this, we compared the TIDE [33] score and levels of immune checkpoints between the two groups, and surprisingly, we discovered that TIDE score was higher and most of the immune checkpoints were highly expressed in the high-HARM group (Figures 7(d) and 7(e)), implying that hypoxia and angiogenesis contributed to the immunosuppressive microenvironment formation.

### 3.7. Compound Development with High-HARH GC

**Figure 8:**
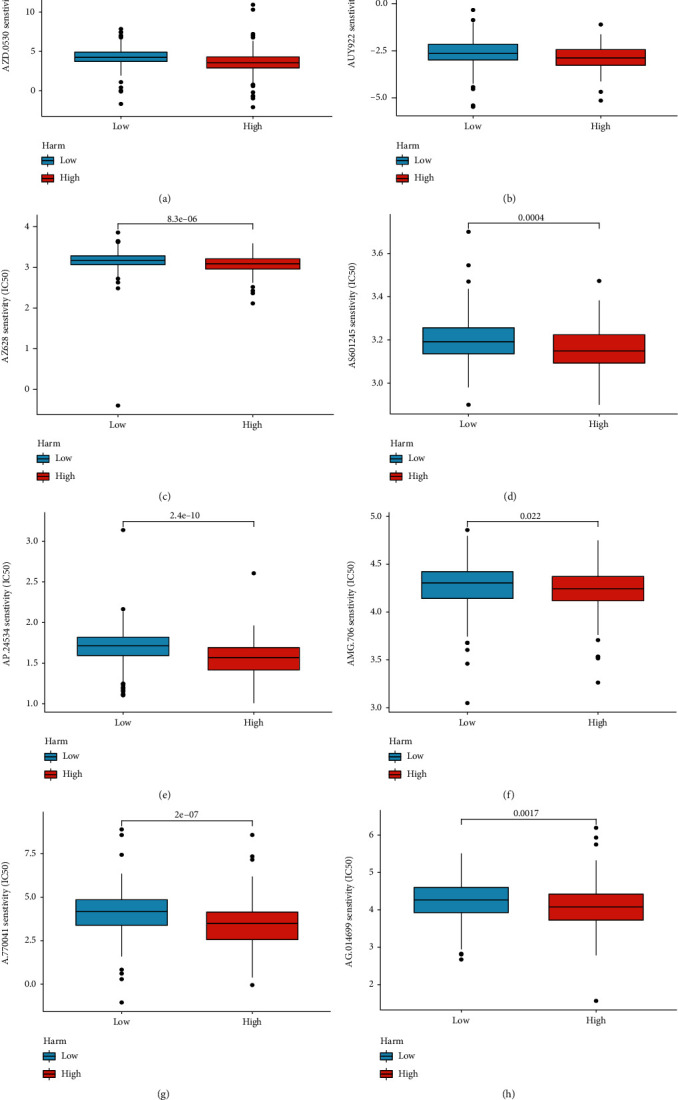
The IC50 differences of the GDSC drugs between the high- and low-HARM groups. (a–h) The box plots displayed the IC50 differences of the 8 GDSC drugs between the HARM groups; the drugs containing AZD.0530 (a), AUY922 (b), AZ628 (c), AS601245 (d), AP.24534 (e), AMG.706 (f), A770041 (g), and AG.014699 (h).

To develop potential drugs against high hypoxia and angiogenesis levels in the GC microenvironment, we then predicted the IC50 of the GDSC compounds against the GCs in both the low- and high-HARM groups. And the top8 compounds are presented in Figures 8(a)–8(h), and all these compounds showed lower IC50 in the high-HARM group.

### 3.8. Single HARM lncRNA Exhibits Clinical Significance in GC Patients

**Figure 9:**
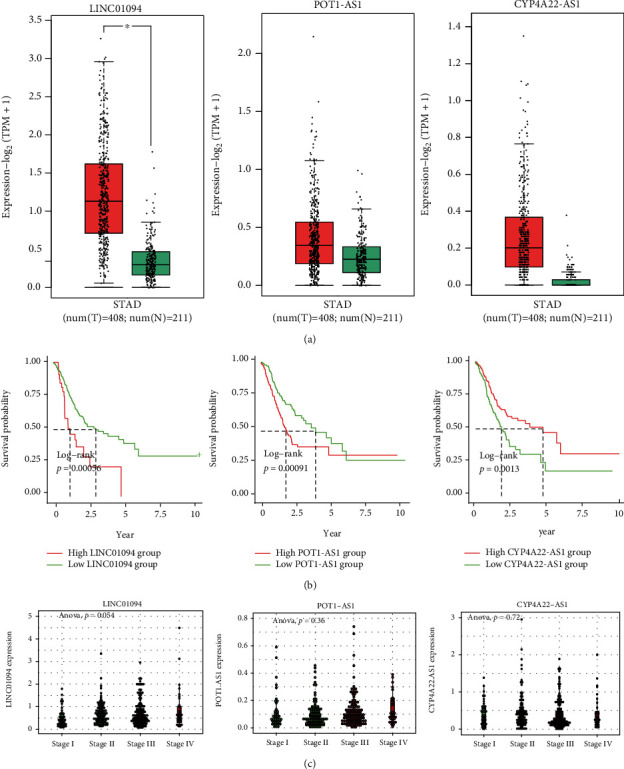
The clinical significance of the single lncRNA in the HARM. The figures presented the expression differences between cancer and noncancer tissues (a), prognostic significance in predicting survival rates (b), and the expression in different stages (c) of LINC01094, POT1-AS1, and CYPA22-AS1 in GCs.

Finally, we investigated the clinical significance of the single lncRNA in the HARM. The results showed that LINC01094 was significantly upregulated in cancers, and POT1-AS1 was also upregulated, though without statistical significance (Figure 9(a)). High LINC01094 and POT1-AS1 levels predicted a worse prognosis for the prognostic value, and CYPA22-AS1 indicated better survival rates (Figure 9(b)). Moreover, LINC01094 expression seemed increased as the cancer stage grew, though with p-value slightly higher than 0.05 (Figure 9(c)). These results suggested LINC01094, POT1-AS1 potential oncogenes, and CYP4A22-AS1 as possible tumor suppressors.

## 4. Discussion

The intense interaction between hypoxia and angiogenesis collectively contributes to the GC progression. This study takes it as the starting point and builds a robust HARM prognostic model for predicting GC patient survival. The accuracy for long-term survival prediction of our HARM model reached 0.783, which was near high discrimination according to the criteria we previously proposed [36]. Moreover, when the HARM was integrated with age and stage, the accuracy for predicting short-term survival was improved, demonstrating the critical roles of hypoxia and angiogenesis in affecting tumor fate. Previously, solo hypoxia or angiogenesis risk scores were constructed [18, 35], but our model outperformed them in survival prediction accuracy, which suggested that the roles of hypoxia and angiogenesis in GC should be considered simultaneously than singly.

The interplay between hypoxia and angiogenesis has been widely proved. Hypoxia condition has been found to promote endothelial cells, vessel formation by exosome reassembly [37], and regulatory T cell employment [38], and direct causes of angiogenesis by hypoxia was mainly attributed to the upregulated VEGF and reprogramming of cancer cells [39]. lncRNA was recently discovered as a bridge that connects upstream hypoxia genes and downstream targets to activate angiogenesis. RAB11B-AS1 was activated by HIF-2*α* under hypoxia and interacted with RNA polymerase II to enhance the proangiogenic gene expression [39]; also, the SNHG1 can be upregulated by HIF-1, and it bound to microRNA-199a-3p to promote TFAM expression for subsequent angiogenesis [40]. However, few studies concerning the involvement of lncRNA in GC hypoxia-angiogenesis interplay were reported. Our study first developed a lncRNA-based hypoxia-angiogenesis signature for GC, and we found a strong association between hypoxia, angiogenesis, and the high-HARM group. This will benefit GC therapy development, and the therapies targeting the “vicious cycle” between hypoxia and angiogenesis have shown promise [41].

For the nine lncRNAs in the HARM, no study of AC090907.1, AC090204.1, or AP00034193 has been reported so far, and our study firstly discovered their prognostic value in cancer. The remaining five lncRNAs (AC019080.5, POT1-AS1, AP000695.2, AP000695.1, and LINC01094) and CYP4A22-AS1 were identified as risky oncogenes and a protective factor, respectively, and this was consistent with the recent findings [42, 43, 44, 45, 46]. And particularly, LINC01094 has been experimentally validated in various cancers to promote their progress [43, 47, 48, 49, 50], demonstrating the high prognostic significance of HARM lncRNAs.

Interestingly, 5 HARM lncRNAs (CYP4A22-AS1, AC019080.5, AP000695.2, AP000695.1, and LINC01094) presented associations with immune in recently published studies [42, 43, 51, 52, 53], suggesting the roles of HARM in cancer immunity modulation. Similarly, we found the high-HARM group was correlated to higher immune infiltration. Notably, M2 macrophages and CAFs were highly infiltrated in this group. Hypoxia can drive the M2 polarization of macrophages in the tumor microenvironment, and the M2 macrophage can, in turn, promote angiogenesis [54], and this modulation has also been elicited in GC [25, 55]. CAFs can trigger angiogenesis under the stimulation of hypoxia-derived extracellular vesicles [56], and hypoxia also activated CAFs' immunosuppressive roles on T cells [57]. In GC, CAFs were found to promote GC cell proliferation under hypoxia [58], while currently, no angiogenetic role of CAFs is discovered in GC. Hence, our study indicated that during hypoxia and angiogenesis, several immunocytes were employed to support cancer growth, and we first suggested the effects of hypoxia-angiogenesis on the association between CAFs and angiogenesis in GC. Besides, the globally increased expression of immune checkpoints in the high-HARM group strongly implied the immunosuppressive roles of the infiltrated immunocytes associated with hypoxia and angiogenesis. Current studies have presented only a few clues about the association between immune checkpoints and angiogenesis/hypoxia. For instance, PD-L1 expressed on tumor-associated macrophages induced T cell apoptosis via binding to PD-1, which was induced by hypoxia [59] in multiple cancer types. Anti-CD40 or anti-VEGFA treatment independently promoted proinflammatory macrophage skewing, and their combination prompted the anticancer function of CD8+ T cells [60]. These suggested HARM's correlations with immune checkpoints and indicated the potential of united therapy of anti-HARM and immune checkpoint blockage; this is novel that no clinical trial concerning any of the HARM lncRNA was carried on. Additionally, since the current evidence is limited, our study first implied the association between hypoxia/angiogenesis and the numerous immune checkpoints in GC.

## 5. Conclusions

Comprehensively, we built a robust HARM lncRNA prediction model by WGCNA and random forest regression and confirmed its high clinical significance for GC patients. The functional analyses indicated that the HARM signature was involved in cancer-associated pathways and macrophage-related immunity regulations. Sequential immunological analyses discovered high infiltration of M2 macrophages and CAFs in the high-HARM group, and immune checkpoints were globally upregulated in this group. Finally, the IC50 of potential HARM-targeted drugs was calculated. This study allowed for novel insights into the interplay between hypoxia-angiogenesis and immunosuppression in GC and will provide novel targets for immunotherapy of GC patients.

## Figures and Tables

**Table 1:** The clinical data of the GC patients from the TCGA dataset.

Characteristics	Alive (*n* = 321)	Dead (*n* = 82)	Total (*n* = 403)
Gender			
Female	123 (38.3%)	22 (26.8%)	145 (36.0%)
Male	198 (61.7%)	60 (73.2%)	258 (64.0%)
Age (mean (SD))	64.9 (10.8)	67.9 (10.4)	65.5 (10.8)
Age (median [min, max])	66 [30, 90]	69 [41, 90]	67 [30, 90]
Stage			
Stage I	44 (13.7%)	11 (13.4%)	55 (13.6%)
Stage II	118 (36.8%)	9 (11.0%)	127 (31.5%)
Stage III	138 (43.0%)	42 (51.2%)	180 (44.7%)
Stage IV	21 (6.5%)	20 (24.4%)	41 (10.2%)

## Data Availability

The sequencing and clinical data used in this study can be obtained from the TCGA-STAD project of TCGA dataset (https://xenabrowser.net/), the gene sets, GO, and KEGG terms can be retrieved from the MSigDB of GSEA (http://www.gsea-msigdb.org/gsea/msigdb/index.jsp), and the R codes can be available from the corresponding author.

## Abbreviations

HARM: Hypoxia-angiogenesis-related model
GC: Gastric cancer
MSI: Microsatellite instability
LncRNA: Long noncoding RNA
WGCNA: Weighted gene coexpression network analysis
MSigDB: Molecular signature database
GSEA: Gene set enrichment analysis
GSVA: Gene set variation analysis
CDF: Cumulative distribution function
PCA: Principal clustering analysis
ROC: Receiver operating characteristics
OS: Overall survival
GO: Gene ontology
KEGG: Kyoto Encyclopedia of Genes and Genomes
IC50: 50% inhibitory concentration
CGP: Cancer genome project
DEG: Differentially expressed genes
ECM: Extracellular matrix
CAF: Cancer-associated fibroblast.
